# Korean Childcare Providers’ Knowledge, Attitudes, Concerns, and Practices of Febrile Convulsions

**DOI:** 10.3390/ijerph18094855

**Published:** 2021-05-02

**Authors:** Won-Oak Oh, Yoo Jin Heo, Min Hyun Suk, Anna Lee

**Affiliations:** 1 School of Nursing, Korea University, Seoul 02841, Korea; wooh@korea.ac.kr (W.-O.O.); witch_22@korea.ac.kr (Y.J.H.); 2 Department of Nursing, College of Health Science, CHA University, Pocheon-si 11160, Korea; chnursing@cha.ac.kr

**Keywords:** early childhood care, childcare providers, febrile convulsions, first-aid practices, fever education

## Abstract

With the increase in the number of childcare facilities, childcare providers’ coping skills to take quick action in emergencies have become crucial. This study was to examine Korean childcare providers’ knowledge, attitudes, concerns, and practices regarding febrile convulsions (FCs), and to identify factors influencing their management of FCs. A cross-sectional study was conducted using a questionnaire of 216 Korean childcare providers. Descriptive statistics were used to describe the sample characteristics and FC questionnaires. The differences in the FC practice by demographic variables were assessed using t-test and one-way analysis of variance. The relationships between FC practice and other variables were investigated using Pearson correlations and regression analysis. The childcare providers showed unfavorable levels of outcomes regarding FCs with a low percentage of correct answers on FC knowledge and recommended practices as well as negative attitudes and concerns toward FCs. Additionally, results indicated that the knowledge, education, attitudes toward, and actual experiences of FCs were related to FC practices. The current findings provide important evidence to develop interventions targeting childcare providers.

## 1. Introduction

In recent years, the number of families using childcare facilities has been steadily increasing [1]. On average, among the Organization for Economic Co-operation and Development Organization (OECD) countries, 32% of children aged 0–2 years are enrolled in early childhood education and care [1]. A report showed that the demand for daycare and early education in the United States (US) has increased dramatically. Approximately 5.1 million children were enrolled in childcare settings in 2019 [2]. In Korea, the number of childcare facilities in 2017 was 39,171, which is 1.5 times an increase from 20,097 childcare facilities in 2001. In total, 1,415,742 children, representing 45.6% of the total child population, used these care facilities in 2018 [3]. The increase in women’s socio-economic activities and more nuclear family types could have contributed to this trend [4,5,6].

Children in childcare facilities should be given more attention with regard to their health issues because their immune system is relatively immature and still has low resistance [7]. Childcare settings in the US and Australia must secure care providers with competent first-aid skills [8]. According to the Enforcement Rule of the Child Care Act of Korea, childcare facilities providing care for 100 or more children must hire a professional nursing staff. However, 66.8% of Korean childcare facilities do not meet the 100-children requirement [9] and most of them do not have a nursing staff. Instead of nurses, childcare providers take the responsibility to provide care for various health issues observed in childcare facilities.

Febrile convulsions (FCs) are one of the most common emergencies faced by childcare providers [10]. The prevalence of FC in Korea among children aged under five years has been reported to be 69.2 in 1000 [11]. Mostly, FCs do not cause severe complications such as long-term mortality and intelligence [12]. However, childcare providers witnessing a child during FC may fear that the child would die or sustain potential brain damage due to insufficient knowledge about FCs and the grotesque manifestation of the convulsions [13,14]. Usually, childcare providers are the first responders to emergencies, including FCs, at childcare centers, although they do not appear to cope well with emergencies [10]. They may hurriedly take children with FCs to emergency rooms without preventing aspiration and keeping the children’s airway clear as the most crucial first-aid treatment for FCs [15].

Appropriate information and education related to children’s health targeting childcare providers have become critical [10,16]. It is necessary to provide appropriate information about FCs to childcare providers who are largely responsible for the health of children in their workplace. Childcare providers’ understanding, perception, or management of FCs should be investigated and discussed.

Most studies regarding Korean childcare centers have predominantly focused on the care available in childcare settings (e.g., how to enhance children’s physical health) but have not considered emergency managements for children in childcare centers [17]. This implies the need for more studies focusing on the emergency management being provided in childcare centers for various health concerns including FCs. Additionally, some previous studies identifying childcare providers’ ability and perception on children’s health and safety issues did not include FCs e.g., [10,18,19,20,21]. Thus, the current study aimed to investigate Korean childcare providers’ knowledge, attitudes, concerns, and practice regarding FCs, and to identify factors influencing their practice for managing children with FCs.

## 2. Materials and Methods

### 2.1. Study Design and Participants

The current study used a cross-sectional survey conducted in March 2019. Participants were recruited from a public educational place in Seoul where childcare providers were gathered for refresher training. The inclusion criteria were childcare providers (1) who understood the survey questionnaires, and (2) who had been working for over six months as childcare providers. Van Voorhis and Morgan (2007) [22] suggested that at least 7 participants (50% power) to 30 participants (80% power) per cell should be considered for the proper sample size when conducting t-test or ANOVA. We estimated a sample size of 215, assuming 13 cells and 15 participants as well as a 10% withdrawal rate.

### 2.2. Instruments

Data were collected through a demographic information questionnaire and another questionnaire developed by Huang et al. [23]. The demographic questionnaire included information about the gender, age, marital status, workplace and position, years of work experience, and experiences of dealing with children with FCs and fever or FC education.

Huang et al. [23] questionnaire is composed of four domains: FC knowledge, attitudes toward FCs, concerns on FCs, and performed first-aid practices during FCs. Huang et al. [23] FC knowledge domain includes 11 items that ask about possible causes of FCs, necessary medical evaluation, risk of FCs recurrence or developing subsequent epilepsy, necessity of anticonvulsants, and recommended/non-recommended practices for seizures, with true/false as well as “don’t know” responses. Some sample items of FC knowledge domain include “anticonvulsant drugs are required for every child with FC,” “recurrent FC will cause brain damage,” and “it is necessary to restrain the child during convulsion.”

In addition to Huang et al. [23] FC knowledge questionnaire, 15 items regarding fever in children were added based on previous research and related literature [7,12,24,25,26]; the items were about definitions, characteristics, management, and knowledge of fever that nursery teachers should have. Total scores were generated by rating correct answers as 1 point and incorrect and “don’t know” answers as no score. Higher scores represent higher levels of knowledge about fever and FC management; the total scores could range from 0 to 26. The internal consistency reliability of the tested instrument was 0.68.

Huang et al. [23] defined attitudes toward FCs as parental opinions about FCs treatment, prognosis, examination, daily care, and relevant sociocultural perspectives. The attitudes domain consists of 10 5-point Likert-scaled items assessing respondents’ attitudes toward FCs. For example, “FC is due to possession by spirits,” “an FC attack is a life-threatening event,” and “it is shameful to have a child with FC.” The total scores could range from 10 to 50, with higher scores indicating somewhat negative perspectives on FCs. The internal consistency reliability of the attitudes domain was 0.64 in this study.

The questionnaire regarding concerns toward FCs contained 10 items scored on a 5-point Likert scale. Some sample items of this domain are “I am worried about further seizure attacks,” “I am worried because I don’t know how to manage during FC attack,” and “I am worried about seizure during sleeping.” The total scores of this domain ranged from 10 to 50, with higher scores representing higher levels of concerns about FCs. The internal consistency reliability of this domain was found to be 0.89.

The practice domain was structured with 14 items covering recommended and non-recommended practices. Participants were asked to check which practice they would use to handle children with FCs, for example, “lower the child’s body temperature” as a recommended practice and “rush the child to a doctor” as a non-recommended practice. Recommended practices were given 1 score while non-recommended practices and “don’t know” responses were given no score. The total scores ranged from 0 to 13, with higher scores implying that a participant tends to use recommended practices toward children with FCs. For the current sample, the internal consistency reliability of the practice domain was 0.73.

### 2.3. Procedure

Before using Huang et al.’s [23] questionnaire, the researchers obtained the permission to use it from the author. The author agreed that the questionnaire could be used to assess childcare providers’ knowledge, attitudes, concerns, and practices regarding FCs. The questionnaire was modified to suit childcare providers (e.g., changing “parents” to “childcare providers”). Then, the items were translated and back translated. The content validity of Huang’s questionnaire and the newly added 15 FC knowledge items was established by an expert panel. The expert panel consisted of six professionals (2 pediatric nurses, 2 nursing professors, 1 pediatric doctor, and 1 childcare provider). Considering the suitability of the items to Korean childcare providers, the panel was asked to evaluate each item. The content validity index at the item level of the 15 items was over 0.80, which indicates that the items had satisfactory content validity.

To solicit participation in this study, the researchers contacted an executive director of the Korean Childcare Providers Association and explained the study’s purpose. Then, the executive director gave permission to introduce the study to childcare providers in a refresher training seminar. Childcare providers who were interested in participating in the current study contacted the investigators directly, and they were given a structured survey consisting of a consent form, a demographic questionnaire, and the questionnaire regarding FCs. The researchers collected the questionnaire back from the participants immediately after they completed filling the questionnaire.

### 2.4. Data Analysis

All analyses were performed with SAS (SAS Institute, Cary, NC, USA). Preliminary data analyses were conducted to assess the reliability of the instrument. The internal consistency of the measure used in this study was identified with Cronbach’s α. Descriptive statistics were used to analyze the characteristics of the sample, demographic data of the participants, and the FC questionnaires. The mean differences of the FC practice according to demographic variables were measured by t-test and one-way analysis of variance. The relationships between the FC practice and other variables were examined using Pearson correlations. To examine the variables that contributed to the FC practice, regression analysis was used. To obtain a parsimonious model, a backward elimination approach was applied to the regression model. The multicollinearity of the final best predicting model was evaluated by a tolerance score. For the final regression model, model assumptions were evaluated using studentized residuals (SRs), including testing for a constant variance with the test of model specification [27], checking for extreme outliers (SRs outside of ±3), and the Shapiro–Wilk (S-W) test for normality. In the current analyses, all the model assumptions of normality were met and there was no multicollinearity issue.

### 2.5. Ethical Considerations

This study was reviewed by the Office of Human Research Ethics at CHA University and this was determined to be an exempt research study. The consent form addressed that data collected would never be used anywhere but for the current study. Further, the participants were assured that they could withdraw from participating in the study anytime. Investigators explained voluntary participation, anonymity, and confidentiality.

## 3. Results

The mean age and standard deviation of the 216 participants whose ages were between 20 and 65 were 42.2 years and 17 respectively (Table 1). Of the 216 participants, 215 were women while one was a man. Most of the participants were married (72.2%) and 63.4% of them had experiences of parenting their children. The majority of the participants had completed formal education with a two-year bachelor degree or higher (82.4%). About 45% of the participants were working at home-based childcare centers and 26.9% are working in private childcare centers. The sample was predominantly staff providers (74.1%). Their years of work experience as childcare providers were quite evenly distributed. Many of them reported that they have observed FC among their family members (78.2%) or children in workplaces (73.2%). Additionally, 68% of them reported that they have cared for children with fever within a year. Although the majority of them expressed the need for fever education (98.1%), more than half of them did not get general fever education (54.2%) or FC education (57.9%).

### 3.1. FC Knowledge, Attitudes, Concerns, and Practices

The mean of the overall FC knowledge score of the current sample was 13.18 (SD = 3.16) and results of subcategories of the knowledge domains are presented in Table 2. The mean of the attitudes toward the FC score, the concern about the FC score, and the FC practice score of the sample were 26.32 (SD = 4.87), 30.33 (SD = 7.92), and 6.77 (SD = 2.57), respectively. Table A1, Table A2, Table A3, Table A4 display the correct answer rates or mean scores of each item.

### 3.2. FC Practices According to Demographic Characteristics

Based on the participants’ reports, there were no significant differences in the FC practice scores by age, marital status, work position, years of work experience, and experiences of caring for children with fever within a year (Table 3). There were statistical differences in the mean scores of FC practice according to the educational level (F = 2.75, *p* = 0.043) and workplaces (F = 6.52, *p* < 0.001) (Table 3). In addition, the mean scores of FC practice varied based on the experiences of observing FC among family members (t = −3.14, *p* = 0.002) or among children in workplaces (t = −3.17, *p* = 0.002), or whether having general fever education (t = −3.65, *p* < 0.001) or FC education (t = −3.69, *p* < 0.001).

### 3.3. Correlations of FC Practices and Other Variables

It was found that the FC practice score was negatively associated with the attitude toward FC score (*p* < 0.001) and positively associated with the seven variables (FC knowledge, marital status, workplaces, experience of observing FCs among family members or children in workplaces, or whether receiving general fever or FC education or not) (Table 4).

### 3.4. Multiple Regression for FC Practices

It was observed that the FC practice was best predicted by FC knowledge, attitudes toward FCs, and having previous fever education or not (Table 5). That is, higher FC knowledge and having fever education led to better FC practice while negative attitudes toward FC led to poor FC practice.

## 4. Discussion

The current study examined Korean childcare providers’ knowledge, attitudes, concerns, and practices regarding FCs, and determined the factors that contributed to their practices for managing children with FCs. Our study demonstrated that childcare providers showed unfavorable levels of outcomes regarding FCs.

The childcare providers participating in this study appeared to have tenuous FC knowledge, with a low percentage of correct answers (57.1%) on the FC knowledge questionnaire. A high proportion of participants had such false knowledge: recurrent FCs will cause brain damage (correct answer rate 7.9%) and putting a protective device into the mouth to prevent tongue injury during convulsion is necessary (correct answer rate 35.6%). They showed better outcomes than parents in a study conducted by Kwak and Kim [28] (correct answer rate 48.5%). However, the current results were inferior to the percentage of parents answering correctly on the FC knowledge in Kayserili et al. [29]’s study (correct answer rate 69.25%). The current level of the FC knowledge among Korean childcare providers should be taken seriously since the role of childcare providers as healthcare supervisor is increasingly demanding [21].

Given that insufficient FC information is an important factor producing anxiety regarding FCs [13], acquiring accurate FC knowledge is crucial to prevent groundless concerns and negative perceptions of FCs. Possibly, the lack of knowledge about FCs leads to false notions and inappropriate management. Failure to handle emergencies appropriately by the childcare centers can have adverse effects on the children in these centers. Additionally, the childcare settings may face negative ramifications regarding the failure to offer proper first-aid responses, which could lead to a lawsuit. Deducing from the above, our results revealed that somewhat negative attitudes and concerns on FCs as well as lower levels of management on children with FCs are obvious among childcare providers.

The present childcare providers appeared to be negative about FCs. For example, the average score of parents who perceived FC attacks as a life-threatening event was 3.49 out of five. The average scores of parents who believed that FC could cause brain damage, and FC could be outgrown were 3.77 and 3.14 out of five, respectively. These results might have been drawn from inaccurate knowledge about FCs given that there is no evidence that FCs cause brain damage and premature death [12].

The current childcare providers had significant concerns on FCs, particularly, “further seizure attack (3.87 out of five)” and “apt to get fever (3.77 out of five).” These results are similar to those reported by previous studies using the same FC concern measurement [23,28], which showed higher levels of concerns on FCs among caregivers with highest concerns on the same items found in the current study.

The present childcare providers described inadequate first-aid practices for handling children with FC. They showed lower levels of FC practices as compared to parents in previous research using the same measurement on examining FC practices [28]. The mean score of FC practice of parents in the study was 9.02 out of 10 and the correct answer rate was 64.4% [28], while those of the current sample were 6.77 and 54.6%. A higher number of childcare providers in this study yielded wrong responses on the following items: “no action when the child showed febrile convulsions” (correct answer rate 19.4%), “try to pry the convulsing child’s clenched teeth apart and put something in his/her mouth” (correct answer rate 23.1%), and “suck discharge from the child’s nose and mouth” (correct answer rate 28.2%). These inadequate coping behaviors toward children with FCs can be hazardous and jeopardizing their safety.

The results of the multiple regression analyses identifying factors influencing the FC practice indicate that FC knowledge and attitude toward FCs, and previously received fever education predicted the practices on FCs. The current results are consistent with previous findings reported by Kwak and Kim [28], which noted that the higher knowledge score for FCs was associated with the higher practice score among 137 caregivers. Similarly, Kanemura et al. [14] found that lower levels of FC knowledge led to more inappropriate FC practice among 84 parents.

Overall, our findings strongly support the importance of FC knowledge. Providing accurate and sufficient information on FCs through proper FC education to childcare providers could enhance their knowledge and practices regarding FCs and alleviate their worries and negative attitudes on FCs, which may have originated from misunderstandings. Despite the importance of the FC education, more than half of childcare providers in this study reported that they had not received education about management of general fever (54.2%) or FCs (57.9%). Moreover, the fact that the majority of childcare providers (67.9%) experienced dealing with children with a fever within a year points to the importance of fever or FC education. Korean childcare providers are regularly trained for health emergency situations. However, the training programs often omit the management of FCs; childcare providers highly desire to learn about FCs [30]. More effort to have Korean childcare providers educated about FCs will be salient.

There is suggestive information drawn from the demographic characteristics for FC education targeting childcare providers. In this study, the participants who have witnessed FC among children within their family or in their workplaces exhibited better practices. This implies that the FC education including hands-on exercises will be effective, which makes childcare providers well prepared for actual situations of FCs. Childcare providers express that even after receiving education, they are still having difficulty in coping properly in actual emergencies. They appeal for more practice-based education than theory-based education on child health problems [21].

## 5. Implications

The current study was the first attempt to assess Korean childcare providers’ knowledge, attitudes, concerns, and practices regarding FCs, and to identify the factors that contributed to their practices for managing children with FCs. This study found the modifiable factors that influence childcare providers’ FC management, such as FC knowledge and attitudes. Researchers could use the current results to develop interventions to enhance the management skills of childcare providers. Childcare providers will benefit from the development of a standard protocol to manage children with FCs. Additionally, the current results present evidence for the need to provide more education for childcare providers. More tailored and specified education programs should be developed for childcare providers.

## 6. Limitations and Recommendations for Future Research

There are some limitations to this study. We used a cross-sectional survey design and examined the outcomes related to FCs using a structured questionnaire. We did not measure their actual practices through observation. Moreover, we recruited participants from restricted areas. Future studies on childcare providers’ management of FCs should consider longitudinal designs, various methods for examining outcomes regarding FCs, and a wide array of participants. Furthermore, future research with qualitative approaches will be warranted to identify a more comprehensive understanding of childcare providers’ management of health emergencies including FCs.

## 7. Conclusions

We found that Korean childcare providers lacked adequate knowledge regarding FCs and had concerns and negative attitudes about FCs. Indeed, they were not conducting optimum first-aid practices. Further, the current findings reveal that FC knowledge and education, as well as actual experiences of FCs, are closely linked to practices regarding FCs. The target education for childcare providers is urgent to improve their knowledge, practice, and concerns on FCs, and to positively change their negative attitudes toward FCs. The present findings could provide an evidence-based intervention and development program for childcare providers.

## Figures and Tables

**Table 1:** General information of participants (*n* = 216).

Variables	Mean ± SD or *n* (%)
Gender	
Female	215 (99.5)
Male	1 (0.5)
Age	42.2 ± 17.0
20–40	79 (37)
41>	137 (63)
Marital status	
Not married	60 (27.8)
Married with parenting experiences	19 (8.8)
Married without parenting experiences	137 (63.4)
Education level	
Institute	38 (17.6)
2-Year college	94 (43.5)
Baccalaureate	70 (32.4)
Graduate	14 (6.5)
Work place	
National childcare center	47 (21.7)
Private childcare center	58 (26.9)
Child center offered by a company	13 (6.0)
Home-based childcare center	98 (45.4)
Work position	
Teacher	160 (74.1)
Senior teacher	18 (8.3)
Deputy head teacher	7 (3.2)
Head teacher	31 (14.4)
Years of work experience ^†^	
6 months–less than 1 year	21 (10.0)
1–5 years	75 (35.0)
6–10 years	64 (30.0)
More than 10 years	55 (25.0)
Observed FCs among family members	
Yes	47 (21.8)
No	169 (78.2)
Observed FCs among children in workplaces	
Yes	58 (26.8)
No	158 (73.2)
Experiences of caring children with a fever during last 1 year ^†^	
Yes	146 (67.9)
No	69 (32.1)
Experience of education regarding general fever management	
Yes	99 (45.8)
No	117 (54.2)
Experience of education regarding FCs	
Yes	91 (42.1)
No	125 (57.9)
Need of education fever or febrile convulsion management ^‡^	
Yes	210 (98.1)
No	4 (2.9)

Note. FCs = febrile convulsions, the sample size was 216 except as otherwise indicated, ^†^ *n* = 215, ^‡^ *n* = 214.

**Table 2:** Main outcomes regarding febrile convulsion (*n* = 216).

Variable	Mean (SD)
Knowledge total	13.18 (3.16)
Fever characteristics	6.59 (1.48)
Fever management	4.25 (1.27)
Febrile convulsion	6.21 (2.14)
Attitude	26.32 (4.87)
Concern	30.33 (7.92)
Practice	6.77 (2.57)

**Table 3:** Febrile convulsion practices according to demographic characteristics (*n* = 216).

Variables	Mean (SD)	t/F	*p*
Age			
20–39	7.32 (2.43)	−1.4	0.16
40–65	7.82 (2.64)		
Marital status			
Not married	7.05 (2.31)	2.21	0.11
Married with parenting experiences	7.79 (2.68)		
Married without parenting experiences	7.88 (2.64)		
Education level			
Institute	7.87 (2.48)	2.75	0.04
2-Year college	7.10 (2.62)		
Baccalaureate	8.21 (2.47)		
Graduate	7.79 (2.46)		
Work place			
National childcare center	6.74 (2.66)	6.52	<0.001
Private childcare center	7.19 (2.17)		
Child center offered by a company	6.85 (2.48)		
Home-based childcare center	8.44 (2.56)		
Work position			
Teacher	7.56 (2.62)	1.65	0.18
Senior teacher	8.17 (2.46)		
Deputy head teacher	6.00 (2.71)		
Head teacher	8.13 (2.28)		
Years of work experience			
Less than 1	7.33 (3.14)	2.33	0.08
1–5 years	7.09 (2.55)		
6–10 years	8.09 (2.40)		
More than 10	8.18 (2.45)		
Observed FCs among family members			
No	7.36 (2.53)	−3.14	0.002
Yes	8.66 (2.49)		
Observed FCs among children in workplaces			
No	7.31 (2.51)	−3.17	0.002
Yes	8.53 (5.23)		
Experiences of caring children with a fever during last 1 year			
No	7.38 (2.71)	−1.00	0.32
Yes	7.75 (2.51)		
Experience of education regarding general fever management			
No	7.06 (2.52)	−3.65	<0.001
Yes	8.31 (2.48)		
Experience of education regarding FCs			
No	7.10 (2.49)	−3.69	<0.001
Yes	8.37 (2.50)		

Note. FCs = febrile convulsions.

**Table 4:** Correlations between febrile convulsion practices and other variables (*n* = 216).

Variables	Practice
Knowledge	0.35 (<0.001)
Attitude	−0.2 (0.003)
Marriage	0.14 (0.043)
Workplaces	0.28 (<0.001)
Observed FCs among family members	0.21 (<0.001)
Observed FCs in workplaces	0.211 (<0.001)
General fever education	0.242 (<0.001)
FC education	0.244 (<0.001)

Note. FCs = febrile convulsions.

**Table 5:** Final regression model of the relationship between febrile convulsion practices and other variables.

Variables	DF	Parameter Estimate	Standard Error	*t*	*p*
Intercept	1	5.83	1.36	4.27	<0.001
FC Knowledge	1	0.24	0.05	4.72	<0.001
FC Attitudes	1	−0.08	0.03	−2.43	0.015
General fever education	1	0.93	0.33	2.88	<0.001

Note. FC = febrile convulsions, the model was significant: F (3,215) = 15.48, *p* < 0.001, DF = degree of freedom, Adj. R^2^ = 0.168.

## Data Availability

The data that support the findings of this study are available from the corresponding author, [AL], upon reasonable request.

## Appendix A

**Table A1:** Percentage of Childcare Providers Answering Correctly on Knowledge about FC attack (*n* = 216).

Item		Correct Answer	Percentage of Answering Correctly *n* (%)
1	Fever refers to when the axillary body temperature is 37.2 degrees Celsius or the tympanic temperature is 38 degrees or higher.	T	152 (70.4)
2	A viral infection is suspected when a child has a fever.	T	125 (57.9)
3	Fever arises from the body’s defense against infection.	T	187 (86.6)
4	Level of fever is closely associated with disease severity.	F	175 (81.0)
5	To measure a 2-year-old child’s temperature with a tympanic thermometer, the earlobe is to be pulled down and back.	T	73 (33.8)
6	The child’s condition or behavior is more important than the degree of fever the child has.	T	123 (56.9)
7	Fever can cause a loss of appetite.	T	177 (81.9)
8	Fever can lead to dehydration.	T	205 (94.9)
9	High fever can cause a febrile seizure.	T	207 (95.8)
10	External cooling methods (such as lukewarm water sponge baths) can increase the child’s body temperature by inducing body shaking.	T	61 (28.2)
11	When child has a fever, I remove most of her/his clothing or keep her/him in lightweight clothing.	T	163 (75.5)
12	Water is not allowed when the child has a fever.	F	193 (89.4)
13	It is very important to lower body temperature by using antipyretics to avoid febrile seizure.	F	62 (28.7)
14	When the temperature does not go down after taking an antipyretic drug, it is useful to give the child an increased dosage.	F	197 (91.2)
15	Antipyretic drug should be taken when body temperature is above 38.3 degrees Celsius.	F	91 (42.1)
16	FC is epilepsy.	F	154 (71.3)
17	Anticonvulsant drugs are required for every child with FC.	F	136 (63.0)
18	Every child with FC will have another FC.	F	79 (36.6)
19	FC is rare after 5.	F	107 (49.5)
20	Recurrent FC will cause brain damage.	F	17 (7.9)
21	Risk of subsequent epilepsy in FC is rare.	T	75 (34.7)
22	It is necessary to put a protective device into the mouth to prevent tongue injury during convulsion.	F	77 (35.6)
23	It is necessary to restrain the child during convulsion.	F	136 (63.0)
24	It is necessary to do mouth-to-mouth resuscitation during convulsion.	F	149 (69.0)
25	Children with FC can receive immunizations on schedule.	T	113 (52.3)
26	EEG or CT is necessary for every child with FC.	F	146 (67.6)
	Total mean percentage of answering correctly		57.1%

Note. FC: Febrile Convulsion; CT: Computerized Tomography; EEG: Electroencephalography; T: Truth; F: False.

**Table A2:** Mean Scores of Items of Childcare Providers’ Concerns about FC attack (*n* = 216).

Items		M(SD)
1	Apt to get fever	3.77 (0.97)
2	Potential brain damage	3.66 (1.09)
3	Subsequent epilepsy	3.27 (1.17)
4	Cannot recognize the seizure attack earlier	3.52 (1.04)
5	Further seizure attacks	3.87 (1.04)
6	FC attack is life-threatening	3.68 (1.12)
7	Don’t know how to manage during FC attack	3.41 (1.1)
8	Delay treatment at next FC attack	3.47 (1.05)
9	Other children will have FC too	2.9 (1.31)
10	Seizure during sleeping	3.56 (1.16)

Note. FC: Febrile Convulsion.

**Table A3:** Mean Scores of Items of Childcare Providers’ Attitude toward FC attack (*n* = 216).

Items		M(SD)
1	FC is due to possession by spirits	1.73 (0.89)
2	FC will become epilepsy	2.63 (1.11)
3	Childcare providers should take their child’s temperature frequently	3.95 (1.0)
4	An FC attack is a life-threatening event	3.49 (1.16)
5	FC can cause brain damage	3.77 (1.01)
6	Folk medicine is also necessary	2.13 (1.0)
7	FC can be outgrown	3.14 (1.04)
8	More attention and care are needed for a child with FC	4.22 (0.88)
9	If necessary, lumbar puncture is acceptable	3.78 (0.91)
10	It is shameful to have a child with FC	1.35 (0.67)

Note. FC: Febrile Convulsion.

**Table A4:** Percentage of Childcare Providers’ Practices during FC Attacks (*n* = 216).

Item		Recommended/Not Recommended Practices	Percentage of Answering Correctly *n* (%)
1	No response	Not recommended	42 (19.4)
2	Shake and rouse the convulsing child	Not recommended	96 (44.4)
3	Cardiac massage	Not recommended	83 (38.4)
4	Protect the child on a soft and safe surface	Recommended	193 (89.4)
5	Stimulate the convulsing child	Not recommended	126 (58.3)
6	Observe seizure manifestations and duration	Recommended	195 (90.3)
7	Rush the child to a doctor	Not recommended	89 (41.2)
8	Restrain the convulsing child	Not recommended	121 (56)
9	Place the child on his/her side	Recommended	168 (77.8)
10	Keep calm	Recommended	147 (68.1)
11	Attempt mouth-to-mouth resuscitation	Not recommended	131 (60.6)
12	Suck discharge from the child’s nose and mouth	Not recommended	61 (28.2)
13	Lower the child’s body temperature	Recommended	148 (68.5)
14	Try to pry the convulsing child’s clenched teeth apart and put something in his/her mouth	Not recommended	50 (23.1)
Total mean percentage of practicing appropriately			54.6%

## References

[1] OECD (2019). Education at a Glance 2019: OECD Indicators. https://www.oecd-ilibrary.org/education/education-at-a-glance-2019_f8d7880d-en.

[2] Grand View Research (2020). U.S. Child Care Market Size & Share Report, 2020–2027. https://www.grandviewresearch.com/industry-analysis/us-child-care-market.

[3] Korea Ministry of Health and Welfare (2019). Statistics on Childcare Facilities and Users. https://www.mohw.go.kr/react/jb/sjb1101vw.jsp?SEQ=103&MENU_ID=03320101&page=1&PAR_MENU_ID=03#.

[4] Flynn L. (2017). Childcare markets and maternal employment: A typology. J. Eur. Soc. Policy.

[5] Kim S.-J., Lee J.-E., Yang S.-O., Kang K.-A., Chang E.Y., Oh K.-S., Seo W.-K., Lee S.-H., Kim S.-H. (2011). Perception of child day care center teachers on issues and needs in child health management. Child Health Nurs. Res..

[6] OECD (2020). Employment Rate.

[7] An H.S., Shin H.Y. (2016). The Pediatrics.

[8] McGrath B.J., Huntington A.D. (2007). The health and wellbeing of adults working in early childhood education. Aust. J. Early Child..

[9] Korea Ministry of Health and Welfare (2012). Early Childhood Care Law. http://www.lawnb.com/lawinfo/contents_view.asp?cid=C39B5D1A899A49FC8C5D08904AD3ADF2|0|K.

[10] Hwang J.Y., Oh E.S., Cho K.J. (2016). A study on the self-confidence in performance and education demand of first aid in kindergarten and daycare center teachers. J. Korea Acad. Ind. Coop. Soc..

[11] Byeon J.H., Kim G.H., Eun B.L. (2018). Prevalence, incidence, and recurrence of febrile seizures in Korean children based on national registry data. J. Clin. Neurol..

[12] Smith D.K., Sadler K.P., Benedum M. (2019). Febrile seizures: Risks, evaluation, and prognosis. Am. Fam. Physician.

[13] Ju H.O., McElmurry B.J., Park C.G., McCreary L., Kim M., Kim E.J. (2011). Anxiety and uncertainty in Korean mothers of children with febrile convulsion: Cross-sectional survey. J. Clin. Nurs..

[14] Kanemura H., Sano F., Mizorogi S., Tando T., Sugita K., Aihara M. (2013). Parental thoughts and actions regarding their child’s first febrile seizure. Pediatr. Int..

[15] Elbilgahy A.A., El Sayed R.A.E.A., El Aziz A. (2018). Effect of implementing an educational module on improving mothers knowledge, home management and attitude about febrile convulsion. J. Nurs. Educ. Pract..

[16] Fetveit A. (2008). Assessment of febrile seizures in children. Eur. J. Pediatr..

[17] Ha K.M. (2020). Conceptualization of Major Stakeholders in Emergency Management of Childcare Facilities. J. Evid.-Based Soc. Work.

[18] Kim H.S., Ra J.S., Lee H.J., Choi E.K. (2008). Health management status of day care center. Child Health Nurs. Res..

[19] Kim I.O., Park H.A. (2012). Survey on knowledge, experience and educational need childcare teacher on infant health management. J. Korea Open Assoc. Early Child. Educ..

[20] Oh K., Sim M.K., Choi E.K. (2008). Knowledge, self-confidence and practice of teachers concerning health and safety of children in child-daycare centers. Child Health Nurs. Res..

[21] Yi S.H. (2019). Effects of First Aid Training Program on Child Care Teachers’ Coping Intentions and Knowledge in the Case of Emergency Situations. J. Korea Contents Assoc..

[22] Van Voorhis C.W., Morgan B.L. (2007). Understanding power and rules of thumb for determining sample sizes. Tutor. Quant. Methods Psychol..

[23] Huang M.C., Huang C.C., Thomas K. (2006). Febrile convulsions: Development and validation of a questionnaire to measure parental knowledge, attitudes, concerns and practices. J. Formos. Med. Assoc..

[24] Walsh A.M., Edwards H.E., Courtney M.D., Wilson J.E., Monaghan S.J. (2005). Fever management: Pediatric nurses’ knowledge, attitudes and influencing factors. J. Adv. Nurs..

[25] Chung Y.S., Oh H.E., Kim J.S. (2010). Parents’ perception, knowledge and self-efficacy in management of childhood fever. Child Health Nurs. Res..

[26] Kim J.S. (2016). Childhood Fever Management: Current Practice vs. Evidence. Child Health Nurs. Res..

[27] White H. (1980). A heteroskedasticity-consistent covariance matrix estimator and a direct test for heteroskedasticity. Econom. J. Econom. Soc..

[28] Kwak A.R., Kim J.S. (2014). Caregivers’ Knowledge, Concerns and Management of Pediatric Febrile Convulsions. Child Health Nurs. Res..

[29] Kayserili E., Ünalp A., Apa H., Asilsoy S., Hizarcioğlu M., Gülez P., Agin H. (2008). Parental knowledge and practices regarding febrile convulsions in Turkish children. Turk. J. Med Sci..

[30] Kim J.H., Heo L.K. (2016). Relationship between Safety Knowledge and Safe Behavior. Korea J. Child Care Educ..

